# Supplementary material for: The Evolution and Characterization of the RNA Interference Pathways in Lophotrochozoa
Source: Genome Biol Evol. 2024 May 7;16(5):evae098. doi: 10.1093/gbe/evae098 (PMC11114477; doi:10.1093/gbe/evae098)
Supplement: evae098_Supplementary_Data [file evae098_supplementary_data.zip › Supplementary_materials.zip!/selected_miRNAs/NoGe_selected_miRNAs/mirdeep_pdf_files/NMRB01001213.1_Notospermus_geniculatus_strain_Ushimado_scaffold1213,_whole_genome_shotgun_sequence_26251.pdf]

Provisional ID	:	NMRB01001213.1_Notospermus_geniculatus_strain_Ushimado_scaffold1213,_whole_genome_shotgun_sequence_26251
Score total	:	21577.7
Score for star read(s)	:	3.9
Score for read counts	:	21569.3
Score for mfe	:	1.5
Score for randfold	:	0
Score for cons. seed	:	3
Total read count	:	42319
Mature read count	:	42195
Loop read count	:	0
Star read count	:	124

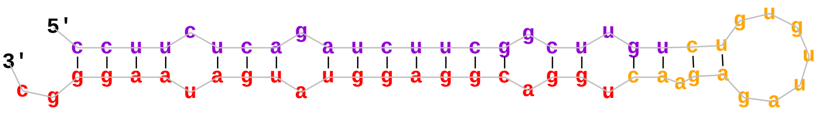

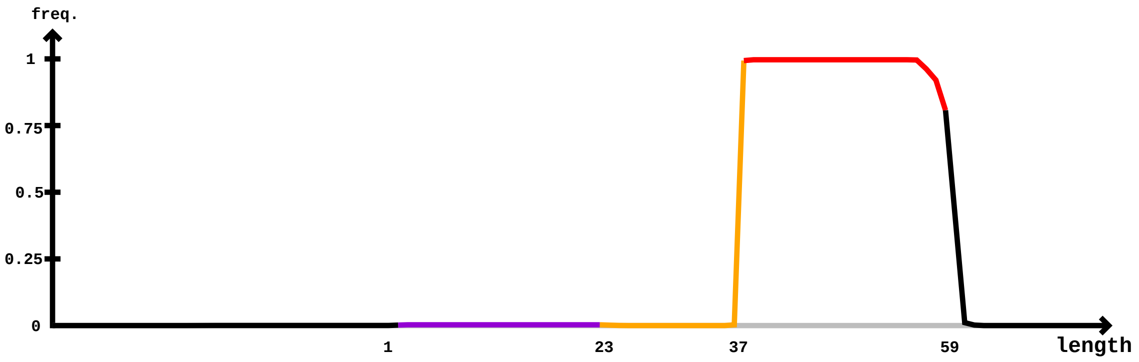

Star **Mature**

```
5'- caucaggagaagagagagaugguuguuugugggcccuucucagaucuucggcuuguguguguuagagaacuggacggagguaugauaagggcuuacugcagcugguac  -3'   obs
    caucaggagaagagagagaugguuguuugugggcccuucucagaucuucggcuugucuguguuagagaacuggacggagguaugauaagggcuuacugcagcugguac         exp
    ((((............))))(((((..((((((((((.(((.(((((((.((.((((.......)).)).)).))))))).))).))))))))))).)))))......   reads   mm   sample
    ..................................ccuucucagaucuucggcuug.....................................................   1       0    Mm3
    ..................................ccuucucagaucuucggcuugu....................................................   6       0    Mm3
    ..................................ccuucucagaucuucggcuuguU...................................................   1       1    Mm3
    ..................................ccuucucagaucuucggcuuguUu..................................................   1       1    Mm3
    ...................................cuucucagaucuucggcuuguc...................................................   4       0    Mm3
    ...................................cuucucagaucuucggcuugucu..................................................   3       0    Mm3
    ...................................cuucucagaucuucggcuugucuU.................................................   1       1    Mm3
    .....................................................................cuggacggagguaugauaaA...................   1       1    Mm3
    .....................................................................cuggacggagguaugauaagg..................   1       0    Mm3
    .....................................................................cuggacggagguaugauaaggg.................   1       0    Mm3
    .....................................................................cuggacggagguCugauaaggg.................   1       1    Mm3
    .....................................................................cuggacggagguaugauaagggc................   1       0    Mm3
    .....................................................................cuggacggagguUugauaagggc................   1       1    Mm3
    ......................................................................uggacggagguaugauaa....................   1       0    Mm3
    ......................................................................uggacggagguaugauaaA...................   3       1    Mm3
    ......................................................................uggacggagguaugauaCg...................   1       1    Mm3
    ......................................................................uggacggagguaugauaUg...................   1       1    Mm3
    ......................................................................uggacggagguaugauaaU...................   1       1    Mm3
    ......................................................................uggacggagguaugauaag...................   35      0    Mm3
    ......................................................................uggacggagguCugauaag...................   2       1    Mm3
    ......................................................................uggacggagguaugauaagg..................   50      0    Mm3
    ......................................................................uggacggagAuaugauaagg..................   2       1    Mm3
    ......................................................................uggacggagguCugauaagg..................   30      1    Mm3
    ......................................................................uggacggagguUugauaagg..................   1       1    Mm3
    ......................................................................uggacggGgguaugauaagg..................   1       1    Mm3
    ......................................................................ugAacggagguaugauaaggg.................   1       1    Mm3
    ......................................................................uggacggagguaugauaaggA.................   6       1    Mm3
    ......................................................................uggacggagguUugauaaggg.................   3       1    Mm3
    ......................................................................uggacggagguaugauaaggU.................   1       1    Mm3
    ......................................................................uggacggUgguaugauaaggg.................   1       1    Mm3
    ......................................................................uggacggaggAaugauaaggg.................   2       1    Mm3
    ......................................................................uggacggagAuaugauaaggg.................   3       1    Mm3
    ......................................................................uggacggagguaugauaGggg.................   1       1    Mm3
```

Star **Mature**

```
caucaggagaagagagagaugguuguuugugggcccuucucagaucuucggcuugucuguguuagagaacuggacggagguaugauaagggcuuacugcagcugguac
......................................................................uggacggagguaugauaaggg.................   207    0   Mm3
......................................................................uggacggagguaugauaUggg.................   3      1   Mm3
......................................................................uggacggagguCugauaaggg.................   22     1   Mm3
......................................................................uggacggagguaugauaagggA................   51     1   Mm3
......................................................................uggacggagAuaugauaagggc................   4      1   Mm3
......................................................................uggacggCgguaugauaagggc................   1      1   Mm3
......................................................................uggacggagguauAauaagggc................   1      1   Mm3
......................................................................uggacggagguaugauaagggG................   2      1   Mm3
......................................................................uggacggaggAaugauaagggc................   9      1   Mm3
......................................................................uggacggagguaugauaagggU................   19     1   Mm3
......................................................................uggacggUgguaugauaagggc................   4      1   Mm3
......................................................................uggacggagguaugauGagggc................   2      1   Mm3
......................................................................uggacggagguaugauaagUgc................   1      1   Mm3
......................................................................uggacggagguaugUuaagggc................   1      1   Mm3
......................................................................ugUacggagguaugauaagggc................   2      1   Mm3
......................................................................uggacggagguaugauUagggc................   5      1   Mm3
......................................................................uggacggagguaugauaGgggc................   7      1   Mm3
......................................................................uggacggagguaugauaUgggc................   7      1   Mm3
......................................................................uggacggGgguaugauaagggc................   1      1   Mm3
......................................................................uggacggagguUugauaagggc................   72     1   Mm3
......................................................................Nggacggagguaugauaagggc................   1      1   Mm3
......................................................................uggacggagguaugauaCgggc................   9      1   Mm3
......................................................................uAgacggagguaugauaagggc................   1      1   Mm3
......................................................................uggacggagguaugauCagggc................   6      1   Mm3
......................................................................Aggacggagguaugauaagggc................   1      1   Mm3
......................................................................uggacggaggGaugauaagggc................   3      1   Mm3
......................................................................uggacggaggCaugauaagggc................   3      1   Mm3
......................................................................uggacggagguCugauaagggc................   15     1   Mm3
......................................................................uggacgUagguaugauaagggc................   1      1   Mm3
......................................................................uggacggagguaugauaagggc................   2049   0   Mm3
......................................................................uggacggagguaugauaCgggcu...............   2      1   Mm3
......................................................................uggacggagguaugauaUgggcu...............   1      1   Mm3
......................................................................uggacggagguUugauaagggcu...............   4      1   Mm3
......................................................................uggacggGgguaugauaagggcu...............   1      1   Mm3
......................................................................uggacggagguaugauaagggcu...............   166    0   Mm3
......................................................................uggacggagguaugauaagggcA...............   96     1   Mm3
......................................................................uggacggagguaugauaagggAu...............   3      1   Mm3
......................................................................uggacggagguaugauGagggcu...............   1      1   Mm3
......................................................................uggacggaggAaugauaagggcu...............   1      1   Mm3
......................................................................uggacggagguCugauaagggcu...............   1      1   Mm3
......................................................................uggacggagguaugauaagggcAu..............   9      1   Mm3
......................................................................uggacggagguaugauaagggcuA..............   1      1   Mm3
......................................................................uggUcggagguaugauaagggcuu..............   1      1   Mm3
......................................................................uggacggagguaugauaagggcuu..............   34     0   Mm3
......................................................................uggacggagguaugauaagggUuu..............   2      1   Mm3
......................................................................uggacggagguaugauaagggcGu..............   1      1   Mm3
......................................................................uggacggagguaugauaagggcuuU.............   17     1   Mm3
.......................................................................ggacggagAuaugauaagg..................   3      1   Mm3
.......................................................................ggacggagguaugauaaggA.................   1      1   Mm3
.......................................................................ggacggagguCugauaaggg.................   2      1   Mm3
.......................................................................ggacggagguCugauaagggc................   1      1   Mm3
.......................................................................ggacggagAuaugauaagggc................   2      1   Mm3
.......................................................................ggacggagguaugauaagggc................   7      0   Mm3
........................................................................gacggagguaugauaagggc................   1      0   Mm3
.........................................................................acggagguaugauaagggc................   1      0   Mm3

...............Aagaugguuguuugugggc..........................................................................   2      1   Fm3
................agaugguuguuugugggc..........................................................................   2      0   Fm3
..................................ccuucucagaucuucggcuug.....................................................   1      0   Fm3
..................................ccuucucagaucuucggcuugu....................................................   12     0   Fm3
..................................ccuucucagaucuucggAuugu....................................................   1      1   Fm3
..................................ccuucucagaucuucggUuugu....................................................   1      1   Fm3
..................................ccuucucagaucuucggcuuguU...................................................   1      1   Fm3
...................................cuucucagaucuucggcuugu....................................................   3      0   Fm3
...................................cuucucagaucuucggcuuguc...................................................   8      0   Fm3
...................................cuucucagaucuucggcuugucu..................................................   10     0   Fm3
.....................................................................cuggacggagguaugauaag...................   5      0   Fm3
.....................................................................cuggacggagguaugaGaag...................   1      1   Fm3
.....................................................................cuggacggagguaugauCagg..................   1      1   Fm3
.....................................................................cuggacggagguaugauaagA..................   2      1   Fm3
```

Star **Mature**

caucaggagaagagagagaugguuguuugugggcccuucucagaucuucggcuugucuguguuagagaacuggacggagguaugauaagggcuuacugcagcugguac			
....cuggacggagguaugauaagg..................	23	0	Fm3
....cuggacggagguaugaGaagg..................	1	1	Fm3
....cuggacggagguaugauaaggg.................	29	0	Fm3
....cuggacggagguaugauaaggA.................	3	1	Fm3
....cuggacggagguaugauaaggC.................	1	1	Fm3
....cuggacggagguaugauaagggA................	11	1	Fm3
....cuggacggagguaugauaagggc................	9	0	Fm3
....cuggacggagAuaugauaagggc................	1	1	Fm3
....cuggacggagguaugauaagggU................	3	1	Fm3
....uggacggagguaugauaa....................	32	0	Fm3
....uggacggagguaugauaU....................	6	1	Fm3
....Nggacggagguaugauaag...................	2	1	Fm3
....uNgacggagguaugauaag...................	1	1	Fm3
....uggacggagguaugaAaag...................	7	1	Fm3
....uggaUggagguaugauaag...................	1	1	Fm3
....uggacggagAuaugauaag...................	6	1	Fm3
....uggacggagguaugauaag...................	969	0	Fm3
....uggacgUagguaugauaag...................	1	1	Fm3
....uggacggaggCaugauaag...................	1	1	Fm3
....uggacggagguCugauaag...................	13	1	Fm3
....uggacggaggAaugauaag...................	5	1	Fm3
....uggacggagguaugauaUg...................	4	1	Fm3
....uggacggagguauAauaag...................	1	1	Fm3
....uggacggagguUugauaag...................	29	1	Fm3
....uggacggaggGaugauaag...................	1	1	Fm3
....uggacggagguaugauaaU...................	43	1	Fm3
....uggacggagguaugauGag...................	1	1	Fm3
....uggacggagguaugauUag...................	1	1	Fm3
....uggacggagguaugauaaA...................	110	1	Fm3
....uggacggagguaugauCag...................	2	1	Fm3
....uggacggagguaugauaCg...................	5	1	Fm3
....uggacggaAguaugauaag...................	4	1	Fm3
....uAgacggagguaugauaag...................	1	1	Fm3
....uggacggagguaugauaGg...................	2	1	Fm3
....uggacggagguUugauaagg..................	19	1	Fm3
....uggacggagguaugauaGgg..................	6	1	Fm3
....uggacggagguaugauaCgg..................	4	1	Fm3
....uggacggagUuaugauaagg..................	1	1	Fm3
....uggacggagguaugauaagU..................	18	1	Fm3
....Nggacggagguaugauaagg..................	1	1	Fm3
....uggacggagguaugauUagg..................	1	1	Fm3
....uggacggagguaugauGagg..................	1	1	Fm3
....uggacggagAuaugauaagg..................	102	1	Fm3
....uggacggagguaugauaUgg..................	2	1	Fm3
....uggacggagguaugauaagA..................	38	1	Fm3
....uggacggagguaugauCagg..................	2	1	Fm3
....uggacggagguaugauaagg..................	893	0	Fm3
....ugUacggagguaugauaagg..................	1	1	Fm3
....ugAacggagguaugauaagg..................	1	1	Fm3
....uggacggagguCugauaagg..................	141	1	Fm3
....uggacggaggCaugauaagg..................	1	1	Fm3
....uggacggaggGaugauaagg..................	1	1	Fm3
....uggacggaggAaugauaagg..................	2	1	Fm3
....uggacgNagguaugauaaggg.................	1	1	Fm3
....uggacggagguaugauaaggU.................	17	1	Fm3
....uggacggagguaugauUaggg.................	4	1	Fm3
....uggacUgagguaugauaaggg.................	2	1	Fm3
....uggacggagguaugauaCggg.................	11	1	Fm3
....uggCcggagguaugauaaggg.................	2	1	Fm3
....uggacggagguaugauaaggg.................	2801	0	Fm3
....uggacggagguaugauCaggg.................	7	1	Fm3
....uggacggagguaugauGaggg.................	4	1	Fm3
....uggacggagguaugauaaggA.................	165	1	Fm3
....uggacggagguaugUuaaggg.................	1	1	Fm3
....uggUcggagguaugauaaggg.................	2	1	Fm3
....uggacggagguaugauaGggg.................	10	1	Fm3
....uggacggagguaugaAaaggg.................	1	1	Fm3
....uggacggaggAaugauaaggg.................	9	1	Fm3
....uggacggagguaugauaaUgg.................	3	1	Fm3
....uggacgUagguaugauaaggg.................	2	1	Fm3

Star Mature

```
caucaggagaagagagagaugguuguuugugggcccuucucagaucuucggcuugucuguguuagagaacuggacggagguaugauaagggcuuacugcagcugguac
......................................................................uggacggagguCugauaaggg..................  109    1  Fm3
......................................................................uggacggagguUugauaaggg..................  28     1  Fm3
......................................................................uggaUggagguaugauaaggg..................  1      1  Fm3
......................................................................Cggacggagguaugauaaggg..................  8      1  Fm3
......................................................................ugUacggagguaugauaaggg..................  1      1  Fm3
......................................................................uggacggagAuaugauaaggg..................  73     1  Fm3
......................................................................uggacggagguaugauaUggg..................  7      1  Fm3
......................................................................Nggacggagguaugauaaggg..................  2      1  Fm3
......................................................................uggacggagguaugauaaAgg..................  2      1  Fm3
......................................................................uggacggaggGaugauaaggg..................  4      1  Fm3
......................................................................uggacggaggCaugauaaggg..................  6      1  Fm3
......................................................................uggacggagguGugauaaggg..................  1      1  Fm3
......................................................................uggacggUgguaugauaaggg..................  2      1  Fm3
......................................................................uggacggGgguaugauaaggg..................  2      1  Fm3
......................................................................uggacggagguaugauaagAgc................  3      1  Fm3
......................................................................uggacNgagguaugauaagggc................  3      1  Fm3
......................................................................uggacggagguaugauaCgggc................  49     1  Fm3
......................................................................uggacggaggCaugauaagggc................  21     1  Fm3
......................................................................uggaGggagguaugauaagggc................  3      1  Fm3
......................................................................uggacggagCuaugauaagggc................  2      1  Fm3
......................................................................uggacggagguaugUuaagggc................  1      1  Fm3
......................................................................uggacggaggGaugauaagggc................  12     1  Fm3
......................................................................ugUacggagguaugauaagggc................  7      1  Fm3
......................................................................ugAacggagguaugauaagggc................  3      1  Fm3
......................................................................uggacggagguaugauaaggCc................  1      1  Fm3
......................................................................uggacggagAuaugauaagggc................  16     1  Fm3
......................................................................uggacAgagguaugauaagggc................  1      1  Fm3
......................................................................uggacggagUuaugauaagggc................  3      1  Fm3
......................................................................uggacCgagguaugauaagggc................  1      1  Fm3
......................................................................uggacggagguauCauaagggc................  2      1  Fm3
......................................................................uggacUgagguaugauaagggc................  4      1  Fm3
......................................................................uggacggagguaugCuaagggc................  1      1  Fm3
......................................................................uggCcggagguaugauaagggc................  2      1  Fm3
......................................................................uggGcggagguaugauaagggc................  2      1  Fm3
......................................................................uggacggCgguaugauaagggc................  2      1  Fm3
......................................................................uCgacggagguaugauaagggc................  3      1  Fm3
......................................................................uggacggGgguaugauaagggc................  1      1  Fm3
......................................................................uggacggagguaugaAaagggc................  2      1  Fm3
......................................................................uUgacggagguaugauaagggc................  3      1  Fm3
......................................................................uggacggagguUugauaagggc................  327    1  Fm3
......................................................................uAgacggagguaugauaagggc................  4      1  Fm3
......................................................................uggacggagguaugauaagggU................  200    1  Fm3
......................................................................uggacggagguaugauGagggc................  14     1  Fm3
......................................................................uggacgCagguaugauaagggc................  1      1  Fm3
......................................................................uggacggagguaugauaagCgc................  1      1  Fm3
......................................................................uggacgNagguaugauaagggc................  1      1  Fm3
......................................................................uggacggagguaugauaaggAc................  5      1  Fm3
......................................................................uggaUggagguaugauaagggc................  2      1  Fm3
......................................................................ugCacggagguaugauaagggc................  1      1  Fm3
......................................................................uggacggagguaugauaGgggc................  52     1  Fm3
......................................................................uggacggagguaCgauaagggc................  3      1  Fm3
......................................................................Cggacggagguaugauaagggc................  1      1  Fm3
......................................................................uggacggaCguaugauaagggc................  1      1  Fm3
......................................................................uggacggaUguaugauaagggc................  5      1  Fm3
......................................................................uggacgAagguaugauaagggc................  3      1  Fm3
......................................................................uggacggagguaugauaagggA................  511    1  Fm3
......................................................................uggacggagguaugauaagUgc................  7      1  Fm3
......................................................................uggacggagguaugauaaggUc................  6      1  Fm3
......................................................................uggacggagguCugauaagggc................  38     1  Fm3
......................................................................uggacggagguaugauCagggc................  28     1  Fm3
......................................................................uggacggagguaugaCaagggc................  2      1  Fm3
......................................................................uggUcggagguaugauaagggc................  3      1  Fm3
......................................................................uggacggaggAaugauaagggc................  33     1  Fm3
......................................................................Nggacggagguaugauaagggc................  7      1  Fm3
......................................................................uggacggagguaugauaUgggc................  46     1  Fm3
......................................................................uggacggagguauUauaagggc................  1      1  Fm3
......................................................................uggacggagguGugauaagggc................  4      1  Fm3
......................................................................uggacggagguaugauaaUggc................  4      1  Fm3
......................................................................uggacggUgguaugauaagggc................  8      1  Fm3
......................................................................uggacggagguaugauaagggc................  14016  0  Fm3
```

```
                                  Star                                 Mature
caucaggagaagagagagaugguuguuugugggcccuucucagaucuucggcuuguc uguguuagagaacuggacggagguaugauaagggcuuacugcagcugguac
......................................................................uggacggagguaugauUagggc................ 14 1 Fm3
......................................................................uggacgUagguaugauaagggc................ 4 1 Fm3
......................................................................uggacggagguauAauaagggc................ 1 1 Fm3
......................................................................uggacggagguCugauaagggcu............... 2 1 Fm3
......................................................................uggacggagguaugauaagggcG............... 4 1 Fm3
......................................................................uggGcggagguaugauaagggcu............... 1 1 Fm3
......................................................................uggacggagguaugauaCgggcu............... 2 1 Fm3
......................................................................uggacggagguaugauaagggAu............... 10 1 Fm3
......................................................................uggacggagguaugauaUgggcu............... 3 1 Fm3
......................................................................uggacggagguaugauaagggcu............... 560 0 Fm3
......................................................................uggacggagguaugUuaagggcu............... 1 1 Fm3
......................................................................uggacggagUuaugauaagggcu............... 1 1 Fm3
......................................................................uggacggagguaugauCagggcu............... 2 1 Fm3
......................................................................uggacggagguaugauaagggUu............... 28 1 Fm3
......................................................................uggacggagguUugauaagggcu............... 8 1 Fm3
......................................................................uggacggaggGaugauaagggcu............... 1 1 Fm3
......................................................................uggacggagguaugauaaggUcu............... 1 1 Fm3
......................................................................uggacggagguaugauUagggcu............... 3 1 Fm3
......................................................................uggacggagguaugauaagggcA............... 830 1 Fm3
......................................................................uggacggagguaugauaagggcuu.............. 23 0 Fm3
......................................................................uggacggagguaugauaagggcuA.............. 7 1 Fm3
......................................................................uggacggagguaugauaagggAuu.............. 4 1 Fm3
......................................................................uggacggagguaugauaagggcCu.............. 1 1 Fm3
......................................................................uggacggagguaugauaagggcAu.............. 47 1 Fm3
......................................................................uggacggagguaugauaagggUuu.............. 2 1 Fm3
.......................................................................ggacggagguCugauaag................... 1 1 Fm3
.......................................................................ggacggagAuaugauaagg.................. 17 1 Fm3
.......................................................................ggacggagguCugauaagg.................. 9 1 Fm3
.......................................................................ggacggagCuaugauaagg.................. 1 1 Fm3
.......................................................................ggacggagguaugauaaggg................. 6 0 Fm3
.......................................................................ggacggagAuaugauaaggg................. 10 1 Fm3
.......................................................................ggacggagguCugauaaggg................. 4 1 Fm3
.......................................................................ggacggagguaugauaaUgg................. 1 1 Fm3
.......................................................................Ugacggagguaugauaagggc................ 1 1 Fm3
.......................................................................ggacggagguaugauaagggA................ 2 1 Fm3
.......................................................................ggacggagguaugauaagggc................ 10 0 Fm3
.......................................................................ggacggagAuaugauaagggc................ 3 1 Fm3
.......................................................................ggacggagguCugauaagggc................ 2 1 Fm3
.......................................................................ggacggagguUugauaagggcu............... 1 1 Fm3
.......................................................................ggacggagguaugauaagggcu............... 2 0 Fm3
.......................................................................ggacggagguaugauaagggcA............... 1 1 Fm3
.......................................................................ggacggagguaugauaagggcAu.............. 1 1 Fm3
..........................................................................cggagguaugauaagggcu............... 1 0 Fm3

..................................ccuucucagaucuucggcuugu.................................................... 24 0 Mm1
......................................................................uggacggagguaugauaCg................... 1 1 Mm1
......................................................................uggacggagguUugauaag................... 1 1 Mm1
......................................................................uggacggagguCugauaag................... 1 1 Mm1
......................................................................uggacggagguaugauaag................... 12 0 Mm1
......................................................................uggacggagguaugauaaA................... 8 1 Mm1
......................................................................uggacggagguaugauaUgg.................. 1 1 Mm1
......................................................................uggacggagguauAauaagg.................. 1 1 Mm1
......................................................................uggCcggagguaugauaagg.................. 1 1 Mm1
......................................................................uggacggagguUugauaagg.................. 1 1 Mm1
......................................................................uggacggagguaugauaagg.................. 134 0 Mm1
......................................................................uggacggagAuaugauaagg.................. 9 1 Mm1
......................................................................uggacggagguaugauCagg.................. 1 1 Mm1
......................................................................uggacggagguCugauaagg.................. 15 1 Mm1
......................................................................uggacggagguaugauaUggg................. 2 1 Mm1
......................................................................uggacggagguaugauaCggg................. 2 1 Mm1
......................................................................uggacggagguaugauaaggg................. 228 0 Mm1
......................................................................uggacggagguaugauGaggg................. 1 1 Mm1
......................................................................uggacggaggAaugauaaggg................. 1 1 Mm1
......................................................................uggacggagAuaugauaaggg................. 2 1 Mm1
......................................................................uggacggagguaugauaaggA................. 27 1 Mm1
......................................................................uggacggagguaugauaaggU................. 2 1 Mm1
......................................................................uggacggaggGaugauaaggg................. 1 1 Mm1
......................................................................uggacNgagguaugauaaggg................. 1 1 Mm1
......................................................................uggacggagguCugauaaggg................. 14 1 Mm1
......................................................................uggacggagguaugauaGggg................. 1 1 Mm1
```

Star Mature

caucaggagaagagagagagaugguuguuugugggcccuucucagaucuucggcuugucuguguuagagaacuggacggagguaugauaagggcuuacugcagcugguac

....uggacggagAuaugauaagggc................	3	1	Mm1
....uggacggagguaugauaagggU................	10	1	Mm1
....uggacggagguaugauaagggc................	2832	0	Mm1
....uggacggagguaugauaagggA................	66	1	Mm1
....uggacggagguauAauaagggc................	1	1	Mm1
....uggaAggagguaugauaagggc................	2	1	Mm1
....uggacggagguaugauaaUggc................	1	1	Mm1
....uggacggagguaugauaagAgc................	1	1	Mm1
....uggacggagguCugauaagggc................	7	1	Mm1
....uggacggagguaugauGagggc................	2	1	Mm1
....uggacgUagguaugauaagggc................	3	1	Mm1
....uggacggagCuaugauaagggc................	1	1	Mm1
....Nggacggagguaugauaagggc................	3	1	Mm1
....uggacggagguaugauUagggc................	2	1	Mm1
....uggacggagguaugauaCgggc................	9	1	Mm1
....uAgacggagguaugauaagggc................	5	1	Mm1
....uggacggaggGaugauaagggc................	2	1	Mm1
....uggacggagguUugauaagggc................	34	1	Mm1
....uggacggagguaCgauaagggc................	2	1	Mm1
....uggacggCgguaugauaagggc................	1	1	Mm1
....uggacggaggCaugauaagggc................	3	1	Mm1
....uggacUgagguaugauaagggc................	1	1	Mm1
....uggacggagguaugauaUgggc................	10	1	Mm1
....uggacNgagguaugauaagggc................	1	1	Mm1
....uggacggagguaugauaGgggc................	10	1	Mm1
....uggacggaggAaugauaagggc................	7	1	Mm1
....uggacggagguaugauCagggc................	3	1	Mm1
....uggacggagguaugauaaggAc................	1	1	Mm1
....uUgacggagguaugauaagggc................	2	1	Mm1
....uggacggagguaugauaagggG................	1	1	Mm1
....uggacggUgguaugauaagggc................	3	1	Mm1
....uggacggagguaugauaGgggcu...............	1	1	Mm1
....uggacggagguaugauaagggUu...............	1	1	Mm1
....uggacggagguUugauaagggcu...............	4	1	Mm1
....uggacggagguaugauaagggcA...............	146	1	Mm1
....uggacggagguaugauaagggcu...............	76	0	Mm1
....uggacggaggAaugauaagggcu...............	1	1	Mm1
....uggacggagguaugauaagggcuu..............	25	0	Mm1
....uggacggagguaugauaagggcAu..............	16	1	Mm1
....uggacggagguaugauaagggcuuU.............	9	1	Mm1
....ggacggagguCugauaag...................	1	1	Mm1
....ggacggagAuaugauaagg..................	1	1	Mm1
....ggacggagguCugauaaggg.................	1	1	Mm1
....ggacggagAuaugauaagggc................	1	1	Mm1
....ggacggagguaugauaagggcu...............	3	0	Mm1
....ggacggagguaugauaagggcuu..............	2	0	Mm1

..........agagaAagaugguuguuugugggc..	1	1	Mm2
................agaugguuguuuguAggc..	1	1	Mm2
..................................ccuucucagaucuucggcuug...	2	0	Mm2
..................................ccuucucagaucuucggcuugu..	10	0	Mm2
..................................ccuucucagaucuucggUuugu..	1	1	Mm2
..................................ccuucucagaucuucggcuuguU...	2	1	Mm2
...................................cuucucagaucuucggcuuguc...	4	0	Mm2
...................................cuucucagaucuucggcuuguU...	2	1	Mm2
...................................cuucucagaucuucggcuugucG..	1	1	Mm2
...................................cuucucagaucuucggcuugucu..	1	0	Mm2
....cuggacggagguaugauaagg..................	2	0	Mm2
....cuggacggagguaugauaagggc................	3	0	Mm2
....uggacggagguUugauaag....................	1	1	Mm2
....uggacggagguaugauaag....................	15	0	Mm2
....uggacggagguCugauaagg...................	5	1	Mm2
....uggacggagguaugauaagg...................	32	0	Mm2
....uAgacggagguaugauaagg...................	1	1	Mm2
....uggacggagguaugauaagA...................	3	1	Mm2
....uggacggaggCaugauaaggg..................	2	1	Mm2
....uggacggagguaugauCaggg..................	1	1	Mm2
....uggacggagguaugauaaggU..................	1	1	Mm2
....uggacggagAuaugauaaggg..................	8	1	Mm2
....uggacggagguCugauaaggg..................	4	1	Mm2

```
                                  Star                                  Mature
caucaggagaagagagagaugguuguuugugggcccuucucagaucuucggcuuguc uguguuagagaacuggacggagguaugauaagggcuuacugcagcugguac
```

```
caucaggagaagagagagaugguuguuugugggcccuucucagaucuucggcuugucuguguuagagaacuggacggagguaugauaagggcuuacugcagcugguac
....................................................................uggacggagguaugauaUggg.................   1      1    Mm2
....................................................................uggacggagguaugauaCggg.................   1      1    Mm2
....................................................................uggacggagguaugauaaggA.................   2      1    Mm2
....................................................................uCgacggagguaugauaaggg.................   1      1    Mm2
....................................................................uggacggagguaugauaagUg.................   1      1    Mm2
....................................................................uggacggagguaugauaaggg.................   149    0    Mm2
....................................................................uggacggagguUugauaaggg.................   2      1    Mm2
....................................................................uggacggagguaugauaaggC.................   1      1    Mm2
....................................................................uggacggagAuaugauaagggc................   6      1    Mm2
....................................................................uggacggagUuaugauaagggc................   1      1    Mm2
....................................................................uggacggUgguaugauaagggc................   1      1    Mm2
....................................................................uggacggagguaugauaaggU................   6      1    Mm2
....................................................................uggacggagguauUauaagggc................   1      1    Mm2
....................................................................uggacNgagguaugauaagggc................   2      1    Mm2
....................................................................uggacggagCuaugauaagggc................   1      1    Mm2
....................................................................uggacggagguaugauaagggc................   1414   0    Mm2
....................................................................uggGcggagguaugauaagggc................   1      1    Mm2
....................................................................uggacggagguaugauaagggG................   1      1    Mm2
....................................................................uggacggagguaugauaCgggc................   6      1    Mm2
....................................................................uggacggagguUugauaagggc................   48     1    Mm2
....................................................................uggacggagguaugauCagggc................   3      1    Mm2
....................................................................uggacggagguaugauaUgggc................   8      1    Mm2
....................................................................uggacggaggCaugauaagggc................   2      1    Mm2
....................................................................Cggacggagguaugauaagggc................   1      1    Mm2
....................................................................uggacggagguaugauaagggA................   11     1    Mm2
....................................................................uggacggagguCugauaagggc................   11     1    Mm2
....................................................................uggacggagguaugauUagggc................   2      1    Mm2
....................................................................uggacggagguaugauGagggc................   1      1    Mm2
....................................................................uggacggaggGaugauaagggc................   1      1    Mm2
....................................................................uAgacggagguaugauaagggc................   1      1    Mm2
....................................................................uggacggaggAaugauaagggc................   4      1    Mm2
....................................................................uggacggagguaugauaaUggc................   1      1    Mm2
....................................................................uggacggagguaugauaGgggc................   5      1    Mm2
....................................................................uggacggagguUugauaagggcu...............   3      1    Mm2
....................................................................uggacggagguCugauaagggcu...............   1      1    Mm2
....................................................................uggacggagguaugauaagggcu...............   69     0    Mm2
....................................................................uggacggagguaugauaagggcA...............   61     1    Mm2
....................................................................uggacggagguaugauaagggcG...............   2      1    Mm2
....................................................................uggacggagguaugauaagggAu...............   1      1    Mm2
....................................................................uggacggaggAaugauaagggcu...............   1      1    Mm2
....................................................................uggacggagguaugauaagggcCu..............   1      1    Mm2
....................................................................uggacggagguaugauaagggcAu..............   12     1    Mm2
....................................................................uggacggagguaugauaagggcuu..............   26     0    Mm2
....................................................................uggaAggagguaugauaagggcuu..............   1      1    Mm2
....................................................................uggacggagguCugauaagggcuu..............   1      1    Mm2
....................................................................uggacggagguaugauaagggcuA..............   2      1    Mm2
....................................................................uggacUgagguaugauaagggcuu..............   1      1    Mm2
....................................................................uggacggagguaugauaagggcuuU.............   8      1    Mm2
.....................................................................ggacggagAuaugauaagg..................   1      1    Mm2
.....................................................................ggacggagAuaugauaaggg.................   2      1    Mm2
.....................................................................ggacggagAuaugauaagggc................   2      1    Mm2
.....................................................................ggacggagguaugauaagggc................   2      0    Mm2

............gaAgaugguuguuugugggc..........................................................................   2      1    Fm1
................................ccuuUucagaucuucggcuugu....................................................   4      1    Fm1
.................................cuuUucagaucuucggcuugu....................................................   2      1    Fm1
.................................cuucucagaucuucggcuuguU...................................................   8      1    Fm1
...................................................................cuggacggagguaugauaagg..................   2      0    Fm1
...................................................................cuggacggagguaugauaaggg.................   2      0    Fm1
...................................................................cuggacggagguaugauaagggc................   9      0    Fm1
....................................................................uggacggagguCugauaag....................   2      1    Fm1
....................................................................uggacggagguaugauaag....................   54     0    Fm1
....................................................................uggacggagguaugauaaA....................   8      1    Fm1
....................................................................uggacggagguUugauaag....................   6      1    Fm1
....................................................................uggacggagguaugauaaU....................   4      1    Fm1
....................................................................uggacggagguaugauaagg...................   85     0    Fm1
....................................................................uggacggagguUugauaagg...................   3      1    Fm1
....................................................................uggacggagguCugauaagg...................   12     1    Fm1
....................................................................uggacNgagguaugauaagg...................   1      1    Fm1
....................................................................uggacggagguaugauaCgg...................   1      1    Fm1
```

```
                                  Star                               Mature
caucaggagaagagagagaugguuguuugugggcccuucucagaucuucggcuuguguguguuagagaacuggacggagguaugauaagggcuuacugcagcugguac
......................................................................uggacggagAuaugauaagg..................  2     1    Fm1
......................................................................uggacggagguaugauaagU..................  1     1    Fm1
......................................................................uggacggagAuaugauaaggg.................  2     1    Fm1
......................................................................uggacggagguaugauUaggg.................  4     1    Fm1
......................................................................uggacggagguaugauaaggA.................  10    1    Fm1
......................................................................uggacggagguaugauaaggU.................  4     1    Fm1
......................................................................uggacggagguaugauaUggg.................  1     1    Fm1
......................................................................uggacggagguaugauaCggg.................  1     1    Fm1
......................................................................uggacggagguaugauaaggC.................  2     1    Fm1
......................................................................uggacggaggAaugauaaggg.................  1     1    Fm1
......................................................................uggacggagguaugauaGggg.................  1     1    Fm1
......................................................................uggacggaggCaugauaaggg.................  2     1    Fm1
......................................................................uggacggagguCugauaaggg.................  15    1    Fm1
......................................................................uggacggagguUugauaaggg.................  1     1    Fm1
......................................................................uggacggagguaugauaaggg.................  460   0    Fm1
......................................................................uggacggagguaugUuaagggc................  1     1    Fm1
......................................................................uggacggagguaugGuaagggc................  2     1    Fm1
......................................................................uggacggagguaugauaagCgc................  1     1    Fm1
......................................................................uggacggagguaugauaUgggc................  21    1    Fm1
......................................................................uggacggagguauUauaagggc................  1     1    Fm1
......................................................................uggaGggagguaugauaagggc................  1     1    Fm1
......................................................................uggGcggagguaugauaagggc................  1     1    Fm1
......................................................................uggacggagguaugauaaggUc................  3     1    Fm1
......................................................................uggacggagguaugauUagggc................  11    1    Fm1
......................................................................uggacggagguaugauaCgggc................  22    1    Fm1
......................................................................uggaAggagguaugauaagggc................  1     1    Fm1
......................................................................uggacggagguaugaCaagggc................  1     1    Fm1
......................................................................uggacggagguCugauaagggc................  41    1    Fm1
......................................................................uggacggaUguaugauaagggc................  2     1    Fm1
......................................................................uggacggagguaugauaGgggc................  32    1    Fm1
......................................................................uggacggaggAaugauaagggc................  9     1    Fm1
......................................................................uggUcggagguaugauaagggc................  1     1    Fm1
......................................................................uggacggCgguaugauaagggc................  2     1    Fm1
......................................................................uggacggagguaugauaagggc................  6213  0    Fm1
......................................................................uggacggagguaAgauaagggc................  1     1    Fm1
......................................................................uggacggaggCaugauaagggc................  4     1    Fm1
......................................................................uggacggagAuaugauaagggc................  8     1    Fm1
......................................................................Cggacggagguaugauaagggc................  1     1    Fm1
......................................................................uggacggagguaugauGagggc................  4     1    Fm1
......................................................................uggCcggagguaugauaagggc................  2     1    Fm1
......................................................................uggacggaggGaugauaagggc................  11    1    Fm1
......................................................................uggacggagguaugauaagggA................  157   1    Fm1
......................................................................uUgacggagguaugauaagggc................  1     1    Fm1
......................................................................uggacggUgguaugauaagggc................  6     1    Fm1
......................................................................uggacNgagguaugauaagggc................  1     1    Fm1
......................................................................uggacggagguaugauCagggc................  15    1    Fm1
......................................................................uggacggagguaugauaaUggc................  2     1    Fm1
......................................................................uggacggagguaCgauaagggc................  2     1    Fm1
......................................................................uggacggagguaugaAaagggc................  1     1    Fm1
......................................................................uggacggGgguaugauaagggc................  3     1    Fm1
......................................................................uAgacggagguaugauaagggc................  3     1    Fm1
......................................................................uggacgCagguaugauaagggc................  2     1    Fm1
......................................................................uggacggagguUugauaagggc................  173   1    Fm1
......................................................................Nggacggagguaugauaagggc................  3     1    Fm1
......................................................................uggacggagguaugauaaggAc................  1     1    Fm1
......................................................................ugAacggagguaugauaagggc................  1     1    Fm1
......................................................................uggacggagguaugauaagggU................  26    1    Fm1
......................................................................ugCacggagguaugauaagggc................  2     1    Fm1
......................................................................uggacggagguaugauaagUgc................  1     1    Fm1
......................................................................uggacggagguaugauaagggG................  4     1    Fm1
......................................................................uggacggagguUugauaagggcu...............  13    1    Fm1
......................................................................uggacggagguaugauaagggAu...............  2     1    Fm1
......................................................................uggacggagguaugauaCgggcu...............  2     1    Fm1
......................................................................uggacggagguaugauaagCgcu...............  1     1    Fm1
......................................................................ugCacggagguaugauaagggcu...............  1     1    Fm1
......................................................................uggacggagguaugauaGgggcu...............  1     1    Fm1
......................................................................Nggacggagguaugauaagggcu...............  1     1    Fm1
......................................................................uggacggagguaugauGagggcu...............  1     1    Fm1
......................................................................uggacggagguaugauaagggcu...............  231   0    Fm1
......................................................................uggGcggagguaugauaagggcu...............  1     1    Fm1
```

```
                                  Star                                 Mature
caucaggagaagagagagagaugguuguuugugggcccuucucagaucuucggcuuguuguguuagagaacuggacggagguaugauaagggcuuacugcagcugguac
```

```
......................................................................uggacggaggGaugauaagggcu...............    1     1   Fm1
......................................................................uCgacggagguaugauaagggcu...............    1     1   Fm1
......................................................................uggacggagguaugauaagggcG...............    5     1   Fm1
......................................................................uggacggagguCugauaagggcu...............    2     1   Fm1
......................................................................uggacggaggAaugauaagggcu...............    2     1   Fm1
......................................................................uggacggagguaugauaagggcA...............    190   1   Fm1
......................................................................uggacggagguaugauaagggcAu..............    31    1   Fm1
......................................................................uggacggagguUugauaagggcuu..............    3     1   Fm1
......................................................................uggacggagguaugauaCgggcuu..............    1     1   Fm1
......................................................................uggacggagguaugauaUgggcuu..............    1     1   Fm1
......................................................................uggacggagguaugauaagggUuu..............    6     1   Fm1
......................................................................uggacggagguaugauaagggcuu..............    70    0   Fm1
......................................................................uggacggagguaugauaagggcuuU.............    20    1   Fm1
.......................................................................ggacggagguCugauaagg..................    1     1   Fm1
.......................................................................ggacggagguaugauaaggg.................    1     0   Fm1
.......................................................................ggacggagguaugauaCggg.................    1     1   Fm1
.......................................................................ggacggagguaugauaagggc................    12    0   Fm1
.......................................................................ggacggagAuaugauaagggc................    1     1   Fm1
.......................................................................ggacggagguaugauaagggcA...............    2     1   Fm1

..............aAagaugguuguuugugggc..........................................................................    6     1   Fm2
..................................ccuucucagaucuucggcuugu....................................................    3     0   Fm2
..................................ccuucucagaucuucggcuuguU...................................................    4     1   Fm2
...................................cuucucagaucuucggcuuguU...................................................    2     1   Fm2
.....................................................................cuggacggagguCugauaagg..................    2     1   Fm2
......................................................................uggacggagguaugauUag....................    1     1   Fm2
......................................................................uggacggaggGaugauaag....................    1     1   Fm2
......................................................................uggacggagguaugauaag....................    48    0   Fm2
......................................................................uggacggagguUugauaag....................    2     1   Fm2
......................................................................uggacggagguaugauaagg...................    47    0   Fm2
......................................................................uggacggagAuaugauaagg...................    4     1   Fm2
......................................................................uggacggagguCugauaagg...................    7     1   Fm2
......................................................................uggacggagguaugauaagU...................    3     1   Fm2
......................................................................uggacggagguaugauaagA...................    6     1   Fm2
......................................................................uggacggagguaugauaaggg..................    204   0   Fm2
......................................................................uggacggagguaugauaUggg..................    1     1   Fm2
......................................................................uggacggagguCugauaaggg..................    14    1   Fm2
......................................................................uggacggagguaugauGaggg..................    1     1   Fm2
......................................................................uggacggagAuaugauaaggg..................    4     1   Fm2
......................................................................uggacggagguaugauaaggA..................    5     1   Fm2
......................................................................uggacggagAuaugauaagggc................    6     1   Fm2
......................................................................uggacggagUuaugauaagggc................    1     1   Fm2
......................................................................uggacgNagguaugauaagggc................    1     1   Fm2
......................................................................uggacggagguaugauaagggA................    37    1   Fm2
......................................................................uggacggaggCaugauaagggc................    2     1   Fm2
......................................................................uggacggagguGugauaagggc................    1     1   Fm2
......................................................................uggacggagguaugauaagggc................    1630  0   Fm2
......................................................................uggUcggagguaugauaagggc................    1     1   Fm2
......................................................................Cggacggagguaugauaagggc................    1     1   Fm2
......................................................................uggacggaggAaugauaagggc................    4     1   Fm2
......................................................................uAgacggagguaugauaagggc................    1     1   Fm2
......................................................................uggacggaUguaugauaagggc................    1     1   Fm2
......................................................................uggacggagCuaugauaagggc................    1     1   Fm2
......................................................................uggacggGgguaugauaagggc................    1     1   Fm2
......................................................................uggacggagguaugauaGgggc................    7     1   Fm2
......................................................................uggacggagguaugauGagggc................    2     1   Fm2
......................................................................uggacggagguaugauaUgggc................    3     1   Fm2
......................................................................uggacggagguCugauaagggc................    15    1   Fm2
......................................................................uggacggagguaugauCagggc................    2     1   Fm2
......................................................................uggacggagguaugauaaUggc................    2     1   Fm2
......................................................................Nggacggagguaugauaagggc................    1     1   Fm2
......................................................................uggCcggagguaugauaagggc................    2     1   Fm2
......................................................................uggacggagguaugauaagggU................    12    1   Fm2
......................................................................uggacggagguaugauaCgggc................    6     1   Fm2
......................................................................uggacggagguUugauaagggc................    48    1   Fm2
......................................................................uggacggagguaugauaagggcG...............    7     1   Fm2
......................................................................uggacggagguCugauaagggcu...............    1     1   Fm2
......................................................................uggacggaggGaugauaagggcu...............    1     1   Fm2
......................................................................uggacggagguaugauaagggcA...............    34    1   Fm2
......................................................................uggacggagguaugauaagggcu...............    72    0   Fm2
```

```
                                 Star                                Mature
caucaggagaagagagagaugguuguuuguggcccuucucagaucuucggcuuguucuguguuagagaacuggacggagguaugauaagggcuuacugcagcugguac
.....................................................................uggacggagguaugauaagggcuu..............   40   0   Fm2
.....................................................................uggacggagguaugauaagggcAu..............   10   1   Fm2
.....................................................................uggacggagguaugauaagggcuuU.............   27   1   Fm2
......................................................................ggacggagguCugauaagg..................   1    1   Fm2
......................................................................ggacggagAuaugauaaggg.................   1    1   Fm2
......................................................................ggacggagguaugauaagggc................   6    0   Fm2
```
